# Predicting 5-Year Mortality After Prosthetic Joint Infection Using a 30-Item Accumulated Frailty Index in a 13,371-Patient Multicenter Cohort

**DOI:** 10.1016/j.artd.2026.102090

**Published:** 2026-09-04

**Authors:** Kole Joachim, Brandon Gettleman, Joshua Wiener, Christopher Hamad, Adrian Lin, Amanda Perrotta, Sumin Jeong, Othneil Sparks, Ezekiel Dingle, Alexandra Stavrakis, Alexander B. Christ

**Affiliations:** a David Geffen School of Medicine at the University of California, Los Angeles, CA, USA; b Department of Orthopaedic Surgery, University of California, Los Angeles, CA, USA; c Case Western Reserve University School of Medicine, Cleveland, OH, USA; d The Center for Clinical Informatics Research and Education, The MetroHealth System, Cleveland, OH, USA; e Department of Orthopaedic Surgery, Greater Los Angeles VA Medical Center, Los Angeles, CA, USA

**Keywords:** Prosthetic joint infection, Mortality, Prediction models

## Abstract

**Background:**

Prosthetic joint infection (PJI) involves a significant mortality risk, with 26-30% of patients dying within 5 years. Current risk assessment tools offer limited prognostic accuracy for PJI patients. This study aimed to develop a prognostic model for 5-year all-cause mortality after PJI using age and frailty assessments.

**Methods:**

We conducted a retrospective cohort study using TriNetX electronic health record data from 13,371 PJI patients diagnosed before 2021. We developed a 30-item Accumulated Frailty Index (AFI-Core30) that incorporates comorbidities, physiologic deficits, medications, and functional status. Generalized additive modeling was used to predict 5-year mortality, with 80% of data allocated for training and 20% for validation. Model performance was assessed using discrimination metrics and calibration techniques.

**Results:**

The mean patient age was 63.1 ± 11.4 years, with an overall 5-year mortality rate of 13.1%. Both AFI-Core30 and age independently predicted mortality (odds ratio per 0.1 AFI increase = 1.84, 95% confidence interval: 1.74-1.95, *P* < .001; odds ratio per 10-year increase in age = 1.65, 95% confidence interval: 1.56-1.74, *P* < .001). The model demonstrated good discrimination (validation area under the receiver operating characteristic curve 0.712) and excellent calibration (Brier score 0.103). Mortality risk ranged from 2.6% in young, nonfrail patients to 50.4% in older, frail individuals.

**Conclusions:**

This validated prognostic model offers the first large-scale, frailty-based tool for predicting mortality in PJI patients. The framework allows for evidence-based risk assessment and personalized treatment planning, shifting PJI prognostication from subjective judgment to objective risk measurement for better clinical decision-making.

## Introduction

Prosthetic joint infection (PJI) is a serious complication following total joint arthroplasty, occurring in approximately 0.25-2% of primary knee arthroplasty (TKA) or total hip arthroplasty (THA), with the overall burden expected to rise as the number of arthroplasties continues to increase [1]. PJI has become the leading cause of early failure in joint replacement, comprising 13-17% of revision surgeries in THA. This imposes a substantial economic burden on the healthcare system, with projected annual hospital costs for PJI in the United States expected to reach $1.85 billion by 2030 [2,3]. Additionally, PJI is linked to high mortality rates, with acute mortality reaching 4% within 90 days of treatment and 1-year mortality rates similarly elevated at 8-11% [1,4]. The long-term prognosis is particularly troubling, as approximately 26%-30% of PJI patients die within 5 years of infection [4,5].

Current research has increasingly focused on identifying patient-specific factors that predict outcomes, especially due to their tendency to outweigh surgical strategy in determining survival after PJI. Advanced age, high comorbidity burden, and frail physiological status consistently predict worse outcomes, yet routine risk assessment still relies mainly on basic measures like ASA class or Charlson scores [5]. Frailty, defined as a cumulative decline in physiologic reserve, better captures vulnerability to stressors such as infection than age alone and has become a strong predictor of perioperative risk in orthopaedic populations [6,7]. For example, the deficit accumulation model produces a continuous Frailty Index (FI) that correlates with an exponential increase in mortality and adverse outcomes, with frail patients experiencing 2-3 times higher postoperative mortality rates in joint arthroplasty [6]. However, current frailty assessment tools offer limited prognostic specificity for PJI patients, lacking calibrated long-term risk estimates that could guide clinical decision-making and patient counseling.

This study aimed to develop a prognostic model for 5-year all-cause mortality following PJI, using age and a 30-item Accumulated Frailty Index (AFI-Core30) as key predictors. Employing generalized additive modeling, we sought to generate individualized risk estimates and create interpretable visual risk maps to enhance shared decision-making and personalized care planning for PJI patients at risk of 5-year mortality.

## Material and methods

### Data source and cohort assembly

We conducted a retrospective cohort study using deidentified electronic health record data from the TriNetX research network. We limited the analysis to patients with PJI diagnoses prior to 2021 to allow for complete 5-year mortality estimates. The arthroplasty cohort included THA and TKA procedures, defined by standard procedure codes listed in Supplemental Table S1. Periprosthetic joint infection was identified with diagnosis codes specific to infection of internal joint prostheses. Nonspecific native-joint infection codes, including septic arthritis codes, were intentionally excluded to maximize diagnostic specificity for PJI and minimize misclassification. The trade-off of modestly reduced case capture was accepted in favor of cohort homogeneity. The final PJI set included adjudicated International Classification of Disease, Version 10 (ICD-10) codes in the T84.5x, T84.6x, and T84.7xx families, and ICD-9 codes 996.66 and 996.67. Diagnoses were linked to arthroplasty by patient identifier, only retaining patients with diagnoses occurring after the index arthroplasty procedure. For patients with multiple qualifying diagnoses, the earliest post-arthroplasty diagnosis was defined as the index PJI.

### AFI-Core30 derivation and scoring

The deficit accumulation approach was selected in accordance with established FI construction methodology, which stipulates a minimum of 30 health deficit variables spanning multiple domains and physiological systems to ensure index reliability and predictive validity [8]. The 30-item threshold was deliberately met to satisfy this standard while remaining constrained to variables extractable from administrative electronic health record data within the TriNetX network.

We constructed the AFI-Core30 using a deficit accumulation approach that incorporates 30 elements. The scoring algorithm generates a continuous measure ranging from 0 to 1 by aggregating individual item scores and dividing by the total number of assessable items for each patient. Data for item assessment were extracted from records documented within 12 months preceding the index PJI date, with the exception of chronic opioid therapy, for which we used a 180-day pre-arthroplasty window.

The index encompasses 3 main categories of deficits. The first category consists of 13 binary comorbidity variables, each assigned a value of 1 when relevant ICD-9-CM or ICD-10-CM diagnostic codes were identified, or 0 in their absence. This group includes immunocompromised states (encompassing HIV/AIDS, congenital immunodeficiencies, patients receiving active cancer treatment, recipients of solid organ transplants, individuals with autoimmune diagnoses, and those prescribed chronic immunosuppressive medications); acute or chronic heart failure; pulmonary disease or asthma of chronic nature; chronic renal disease; diabetes mellitus; coronary artery disease or prior myocardial infarction; atrial fibrillation; cerebrovascular accident or transient ischemic attack history; peripheral arterial disease; hepatic cirrhosis; dementia; depression or anxiety disorders; and current or historical malignancy.

The second category comprises 9 physiological and laboratory-based deficits identified through diagnostic code proxies, with most items scored as 0.5 when documented abnormalities were present and 0 when absent. These include hypoalbuminemia, anemia, sodium dysregulation (encompassing both hyponatremia and hypernatremia), lymphopenia, inadequate glycemic control, heart rate abnormalities, blood pressure deviations (including both hypotension and hypertension), and hypoxemia. Body mass index (BMI) represents a graded exception within this category: underweight status or severe obesity (BMI 40-59 kg/m^2^) received 0.5 points, extreme obesity (BMI ≥60 kg/m^2^) received 1.0 point, and normal or overweight status scored 0.

Medication exposure constitutes the third deficit category, with 2 components. Benzodiazepine or antipsychotic prescriptions were scored dichotomously (1 if documented, 0 if not). Chronic opioid therapy was evaluated over the 180-day pre-PJI period using a graduated scoring system based on prescription frequency as a surrogate for cumulative exposure: absence of prescriptions scored 0, intermittent therapy (fewer than 3 prescriptions, approximating <90 days' supply) scored 0.5, and chronic use (3 or more prescriptions, approximating ≥90 days' supply) scored 1.

Five healthcare utilization and functional status indicators complete the index. Inpatient admissions were graded as 0 for none, 0.5 for a single hospitalization, and 1 for multiple hospitalizations. Emergency department utilization followed a similar pattern: 0 for 0-1 visits, 0.5 for 2-3 visits, and 1 for ≥4 visits. Documented fall events were scored 0 for none, 0.5 for one occurrence, and 1 for multiple falls. Durable medical equipment used for mobility assistance (canes, walkers, or wheelchairs) contributed 0.5 points when documented. Nursing facility residency added 1 point when coded. Finally, unintentional weight loss or diagnosed malnutrition contributed 0.5 if present and 0 if absent. Supplemental Table S2 presents comprehensive ICD-9-CM and ICD-10-CM code mappings, encounter-based definitions, medication coding schemes, and implementation specifications for the AFI-Core30.

### Outcomes and statistical modeling

The primary outcome was 5-year all-cause mortality after the index PJI, defined as death within 1826 days of the infection date. We first described crude mortality by AFI-Core30 categories (nonfrail, prefrail, frail) and by age tertiles. To estimate the independent contributions of frailty and age, we fit a logistic regression with 2 predictors: AFI-Core30 and age at PJI. Results are reported as odds ratios (OR) per 0.1 increase in AFI and per 10-year increase in age with 95% confidence intervals (95% CI), with *P* < .05 considered significant. Regression model selection was supplemented with the Akaike Information Criterion (AIC), where lower AIC values indicate a better model fit. To produce point-of-care risk estimates across the full range of age and AFI-Core30, we used a flexible generalized additive model that allows nonlinear effects. We specified separate smooth terms with 6 splines each for age and AFI-Core30 and omitted an interaction term to ensure model stability. After removing 12 patients with extreme age outliers (<18 years), data were split into 80% for model training and 20% for validation. Predictors were centered and scaled using only the training data, and extreme values were clipped to ±4 standard deviations to prevent outliers from influencing the model. Because deaths were less common than survivals, we applied inverse-prevalence weights capped at 10 to address class imbalance. The smoothing penalty was chosen on the training set using 3-fold stratified cross-validation over a logarithmic grid (10^4^ to 10^8^), with the area under the receiver operating characteristic curve (AUROC) as the selection criterion, and then the model was refitted on the full training set.

### Validation, calibration, and visualization

On the validation set, we assessed discrimination using the AUROC and the area under the precision–recall curve (PR-AUC), and overall accuracy with the Brier score. Calibration was summarized with a 10-bin quantile calibration table. We then calibrated predicted probabilities on the validation set by comparing 3 methods: bounded isotonic regression, Platt scaling, and beta calibration. Isotonic regression was constrained to the minimum and maximum observed event rates across the quantile bins to prevent extrapolation beyond the empirical range. The final calibrator was chosen based on the lowest Brier score, with a tolerance of 0.002 for ties, and preference was given to smoother methods (beta > Platt > isotonic) when performance was similar. Figures were generated from the validated mortality probabilities and included a 2-dimensional heatmap with isoprobability contours, a discrete risk band plot with mortality risk categories of 10%, 10-25%, 25-50%, and greater than 50%, and an interactive 3-dimensional surface plot with the color scale and z-axis limited to the calibrated range. Claude Sonnet 4.5 (Anthropic, San Francisco, CA) was used to refine grammar and phrasing during manuscript preparation. OpenEvidence was utilized as an adjunct tool to assist with citation verification and literature sourcing. The authors affirm that all intellectual content, including study conception, data analysis, interpretation, and the generation of scientific claims or conclusions, was conducted by the authors themselves without the use of generative artificial intelligence. All AI tools were employed solely for language refinement and citation management, and all outputs were independently reviewed and verified by the authors for accuracy and integrity.

## Results

### Cohort and AFI-Core30 distribution

The study cohort included 13,371 patients with PJI occurring before 2021, with a mean follow-up of 9.3 years (range: 4.7-36.4 years) and a mean age at the time of PJI of 63.1 ± 11.4 years. The median time from initial arthroplasty to PJI was 70 days (interquartile range: 19-478 days). Notably, frail patients developed PJI sooner than nonfrail patients (median 41 days vs 195 days, *P* < .001). The mean AFI-Core30 score was 0.144 ± 0.087, with the most common deficits being recent opioid use (81.0%), recent hospitalizations (75.8%), and abnormal blood pressure (73.5%) (Table 1). Overall, 5-year all-cause mortality was 13.1%. Unadjusted mortality analysis showed increased risks across frailty categories (nonfrail 7.1%, n = 5037; prefrail 12.9%, n = 6541; frail 30.2%, n = 1,793, *P* < .001).

**Table 1:** AFI-Core30 Components for PJI patients included in mortality risk analysis stratified by sex.

AFI-Core30 components	Overall (N)	Overall (%)	Female (N)	Female (%)	Male (N)	Male (%)
Recent opioid exposure	10,458	81.0	5363	81.2	5095	80.7
Recent hospitalizations	9784	75.8	4954	75.0	4830	76.5
Abnormal blood pressure	9492	73.5	4852	73.5	4640	73.5
Depression/anxiety	4428	34.3	2744	41.6	1684	26.7
Low/high body mass index	3925	30.4	2108	31.9	1817	28.8
Diabetes mellitus	3236	25.1	1599	24.2	1637	25.9
Immunosuppressed	3127	24.2	1697	25.7	1430	22.7
Low hemoglobin (anemia)	2810	21.8	1586	24.0	1224	19.4
Benzodiazepine/antipsychotic use	2413	18.7	1451	22.0	962	15.2
Coronary artery disease/MI	2301	17.8	924	14.0	1377	21.8
Chronic kidney disease	1960	15.2	914	13.8	1046	16.6
Atrial fibrillation	1813	14.0	770	11.7	1043	16.5
Congestive heart failure	1625	12.6	803	12.2	822	13.0
Low sodium	1570	12.2	791	12.0	779	12.3
COPD/asthma	1565	12.1	877	13.3	688	10.9
Emergency department visits	1465	11.3	802	12.1	663	10.5
Peripheral vascular disease	1034	8.0	464	7.0	570	9.0
Elevated hemoglobin A1c	964	7.5	448	6.8	516	8.2
Abnormal heart rate	915	7.1	477	7.2	438	6.9
Mobility equipment use	719	5.6	348	5.3	371	5.9
Nursing facility residence	569	4.4	294	4.5	275	4.4
Low oxygen saturation	547	4.2	318	4.8	229	3.6
History of falls	430	3.3	266	4.0	164	2.6
Liver cirrhosis	365	2.8	164	2.5	201	3.2
Dementia	284	2.2	159	2.4	125	2.0
Unintentional weight loss	235	1.8	112	1.7	123	1.9
Cancer/malignancy	233	1.8	25	0.4	208	3.3
Low albumin	210	1.6	117	1.8	93	1.5
Stroke/transient ischemic attack	122	0.9	61	0.9	61	1.0
Low lymphocytes	19	0.1	10	0.2	9	0.1

MI, myocardial infarction; COPD, chronic obstructive pulmonary disease.

### Adjusted associations of AFI-Core30 and age with mortality

**Supplemental Figure S1:**
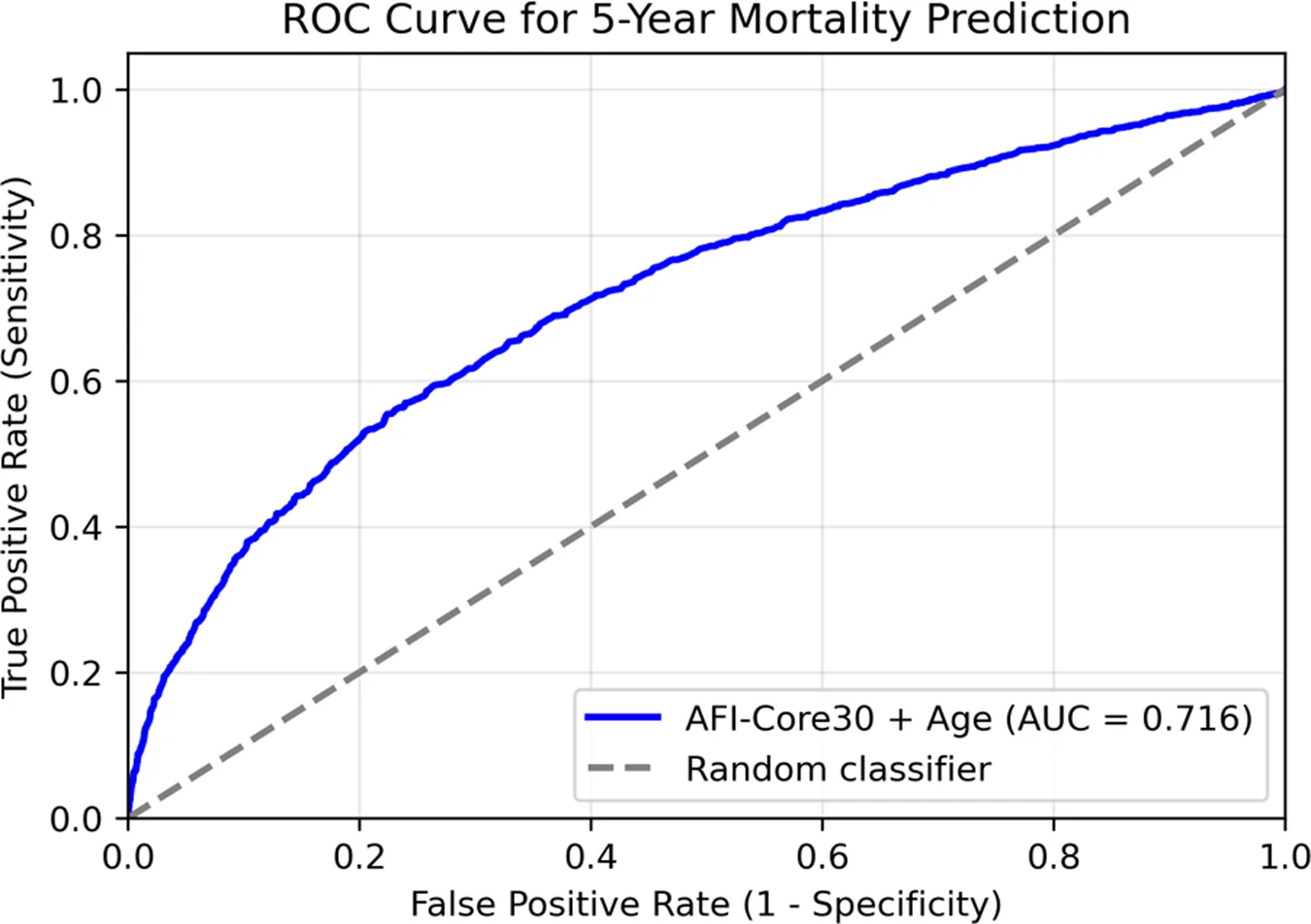


In a logistic regression including AFI-Core30 and age among 12,568 patients with complete data, both predictors were independently linked to 5-year mortality (OR per 0.1 AFI increase = 1.84, 95% CI: 1.74-1.95, *P* < .001; OR per 10-year increase in age = 1.65, 95% CI: 1.56-1.74, *P* < .001) with an AUC of 0.716 (Supplemental Fig. S1). Adding an AFI-Core30×age interaction term did not improve the model fit (ΔAIC = +0.2) and was not significant (OR = 1.04, 95% CI: 0.98-1.11; *P* = .187).

### Predictive performance and calibration of the risk model

**Figure 1:**
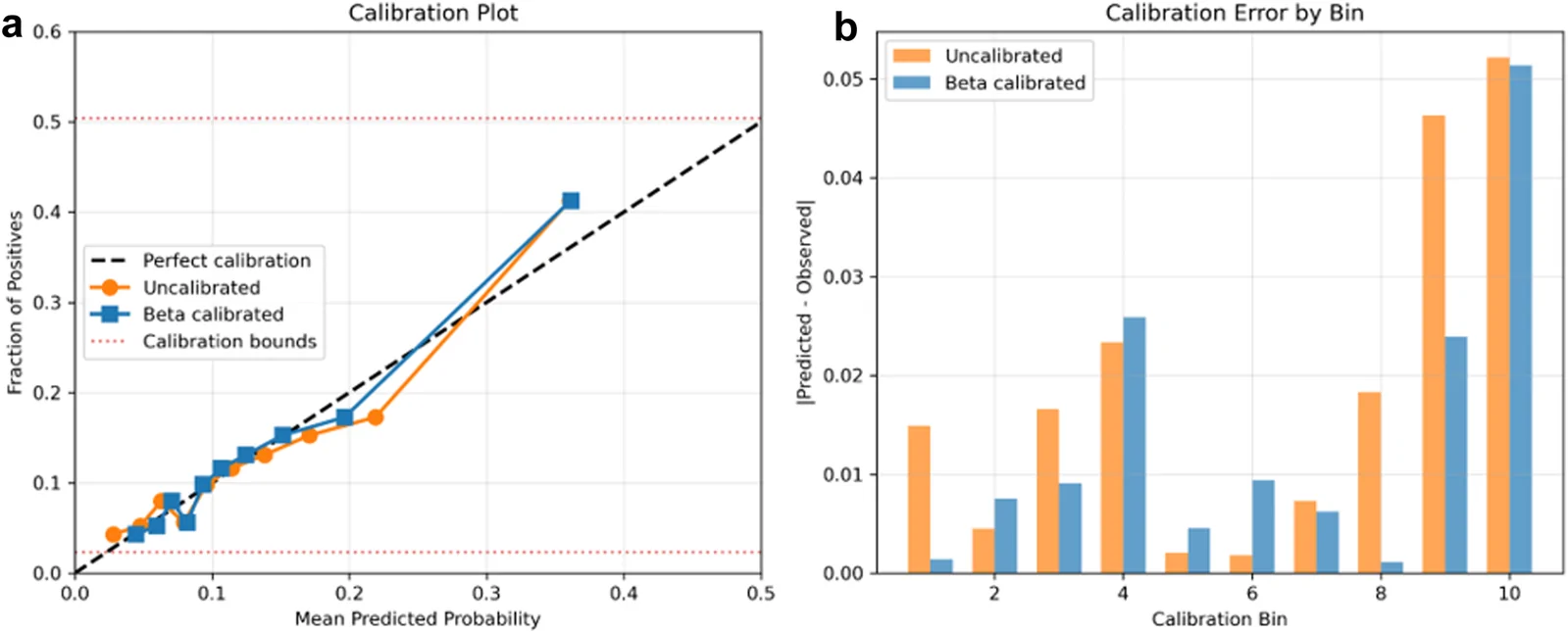
Calibration assessment of AFI-Core30 plus age model. Calibration analysis of the generalized additive model for 5-year mortality prediction in the validation set (N = 2512). (a) Calibration plot comparing uncalibrated and beta-calibrated predictions across 10 quantile bins. The diagonal reference line represents perfect calibration, while horizontal dashed lines show calibration bounds. (b) Absolute calibration error by bin, demonstrating improved alignment between predicted probabilities and observed mortality rates after beta calibration.

On the validation set (N = 2512), discrimination was AUROC 0.712 and PR-AUC 0.333. Overall accuracy measured by the Brier score was 0.103 for predictions. Calibration using 10 equal-frequency bins showed observed event rates ranging from 2.3% to 50.4%, which established the bounds for reporting probabilities (Table 2). Among calibration methods, isotonic regression achieved the lowest Brier score (isotonic 0.102, Platt 0.103, beta 0.103), but beta calibration was chosen for its smoother calibration profile, as performance differences were minimal (<0.002) (Fig. 1). All reported probabilities stayed within the 2.3-50.4% range to prevent extrapolation beyond the validation data.

**Table 2:** Model calibration performance: predicted vs observed 5-year mortality risk after prosthetic joint infection by risk decile.

Bin	Patients	Observed rate	Average predicted (uncalibrated)	Average predicted (calibrated)	Error (uncalibrated)	Error (calibrated)
1	255	0.043	0.028	0.045	0.015	0.001
2	249	0.052	0.048	0.060	0.004	0.008
3	251	0.080	0.063	0.071	0.017	0.009
4	250	0.056	0.079	0.082	0.023	0.026
5	255	0.098	0.096	0.093	0.002	0.005
6	250	0.116	0.114	0.107	0.002	0.009
7	252	0.131	0.138	0.125	0.007	0.006
8	249	0.153	0.171	0.151	0.018	0.001
9	249	0.173	0.219	0.197	0.046	0.024
10	252	0.413	0.361	0.361	0.052	0.051

Model performance showing predicted mortality risk vs actual patient outcomes across 20 risk groups. Calibration adjustment improved prediction accuracy, with final model predictions closely matching observed 5-year mortality rates (range: 2.3% to 50.4%).

### Risk maps and point-of-care examples

**Figure 2:**
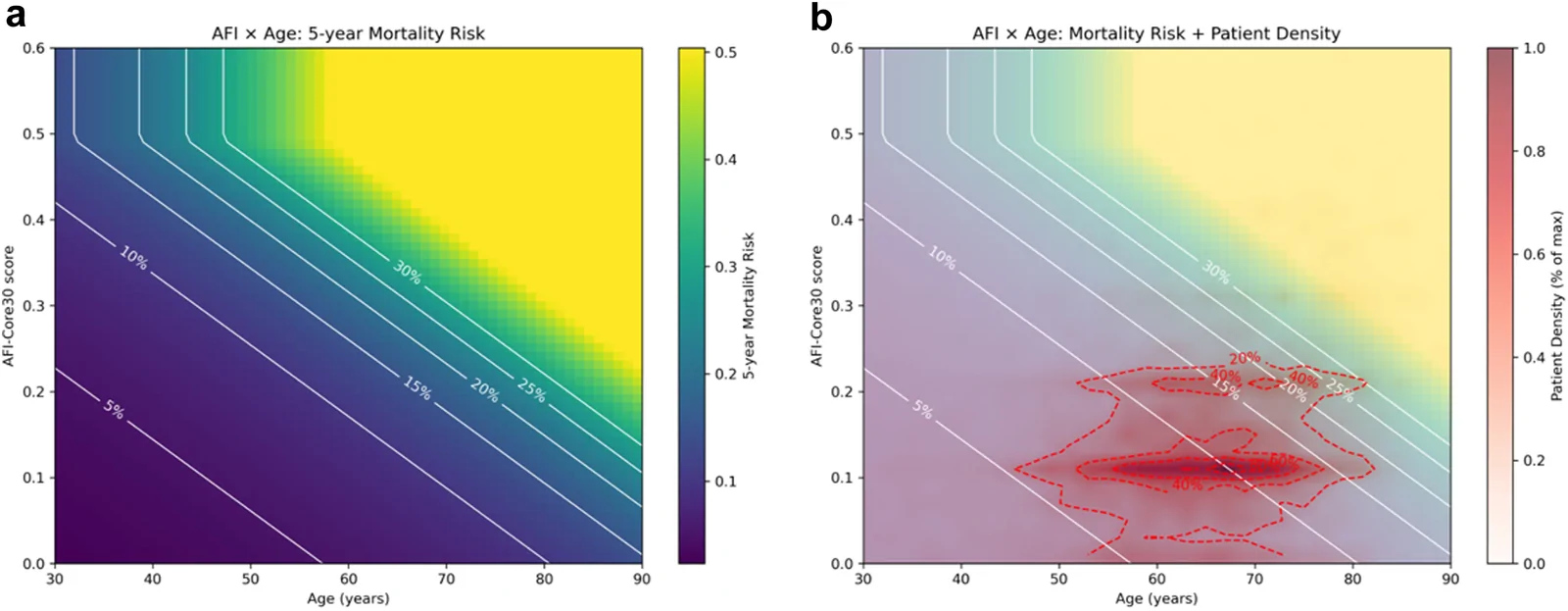
AFI-Core30 and age-stratified 5-year mortality risk after PJI. (a) Calibrated mortality risk surface showing 5-year mortality probability across patient age and AFI-Core30 frailty scores. White contour lines indicate mortality risk percentiles (5%, 10%, 15%, 20%, 25%, and 30%). (b) Same mortality risk surface overlaid with patient density distribution (red contours showing 20%, 40%, 60%, 80%, and 100% of maximum density) for 13,371 patients in the study cohort. The patient density overlay reveals the concentration of actual patients within different age-frailty risk strata, with most patients clustering in lower AFI scores and middle-age ranges.

**Figure 3:**
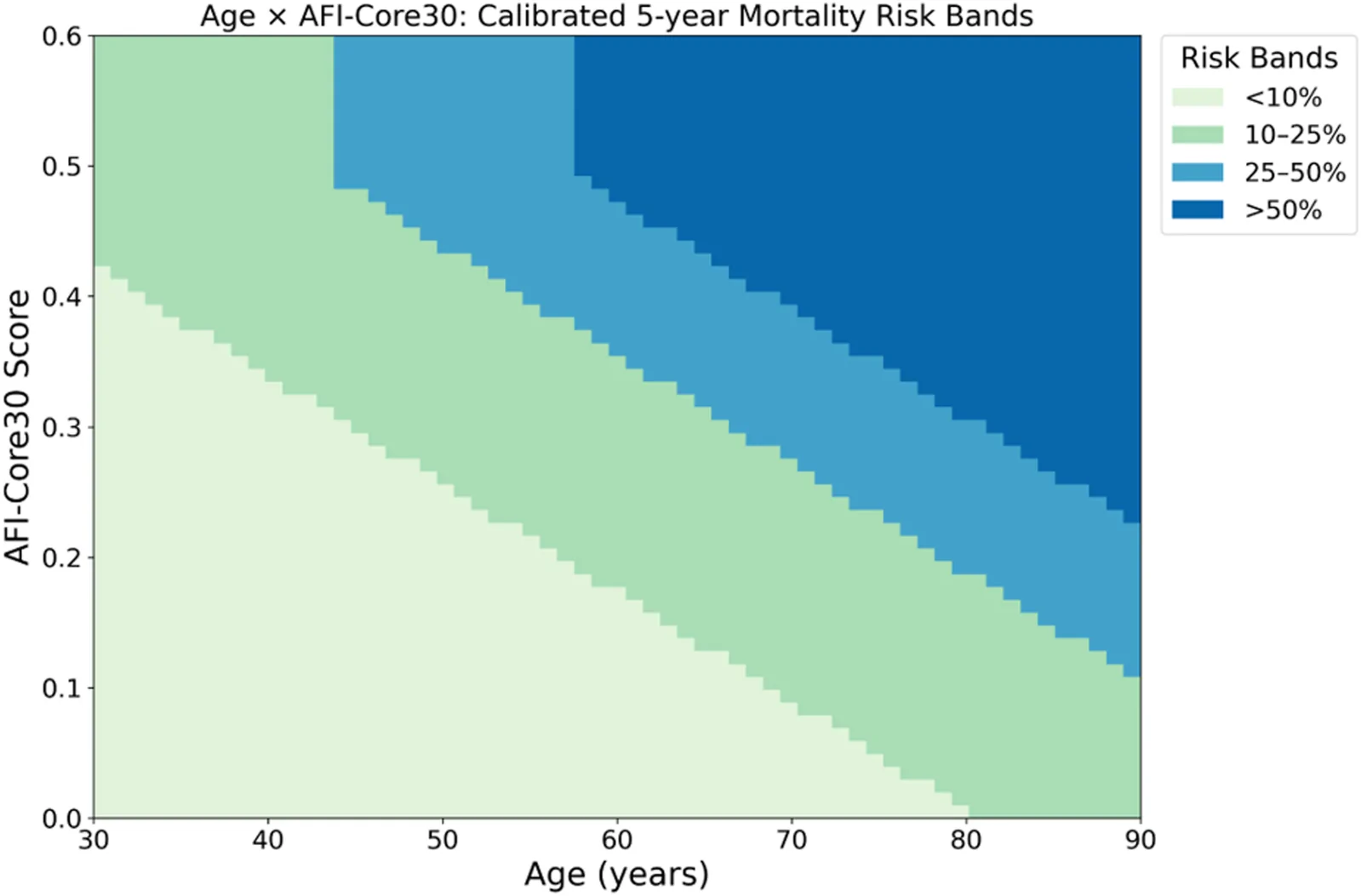
AFI-Core30 and age-stratified 5-year mortality risk bands after PJI. Discrete risk stratification showing calibrated 5-year mortality probability bands across patient age and AFI-Core30 frailty scores. Risk bands are defined as low risk (<10% mortality, light green), moderate risk (10-25% mortality, medium green), high risk (25-50% mortality, light blue), and very high risk (>50% mortality, dark blue).

We generated calibrated absolute risks using the generalized additive model over the observed age and AFI-Core30 ranges. The lowest risk was 2.6% at age 30 with AFI 0.000, while the highest risk was 50.4% at age 90 with AFI 0.230. At age 50, estimated 5-year mortality increased from 4.8% at AFI 0.05 to 8.1% at AFI 0.20, 12.2% at AFI 0.30, 20.1% at AFI 0.40, and 34.4% at AFI 0.55. At age 60, risks were 7.6% at AFI 0.10, 11.3% at 0.20, 18.4% at 0.30, and 32.4% at 0.40, with 50.4% at 0.55. At age 70, risks were 10.5% at AFI 0.10, 16.8% at 0.20, 29.3% at 0.30, and 50.4% at 0.40 or higher. At age 80, risks were 12.2% at AFI 0.05, 15.5% at 0.10, 26.6% at 0.20, 47.4% at 0.30, and 50.4% at 0.40 or higher (Fig. 2A). Patient density across the age-AFI risk surface is shown in Figure 2B. A complementary band plot divides the surface into risk categories of less than 10%, 10-25%, 25-50%, and greater than 50% (Fig. 3). These values are visualized in the accompanying interactive 3-dimensional surface, which maps age and AFI-Core30 to calibrated 5-year mortality risk (Supplemental Fig. S2).

## Discussion

In this retrospective multicenter cohort of 13,371 PJI patients, we showed that patient age and frailty status are significantly predictive of 5-year mortality, with each additional decade of age associated with a 1.65-fold higher odds of mortality and each 0.1 increase in AFI-Core30 score resulting in a 1.84-fold higher mortality odds. The prognostic model combining age and AFI-Core30 achieved a validation AUROC of 0.712 and excellent calibration with a Brier score of 0.103, indicating high accuracy in mortality probability estimates. The risk stratification ranges from 2.6% 5-year mortality in younger, low-frailty patients to 50.4% in high-risk profiles, aligning with previous reports estimating 5-year mortality rates as high as 30% in Medicare PJI analyses [4]. This range supports the clinical usefulness of personalized risk estimation for predicting 5-year mortality in PJI patients.

Our utilization of AFI-Core30 derived from TriNetX data offers greater prognostic granularity than traditional indices such as the Charlson Comorbidity Index (CCI) or the 5-factor modified frailty index (FI), as CCI scores summarize comorbid diseases but omit functional status and other age-related deficits. Meanwhile, 5-factor modified FI relies on 5 binary variables that can saturate in older patients [[9], [10], [11], [12], [13]]. In contrast, our deficit accumulation approach includes a broader range of health deficits, resulting in a continuous frailty score. This method effectively integrates numerous minor deficits that individually do not qualify as comorbidities but collectively indicate reduced physiologic reserve, signifying frailty despite low traditional comorbidity scores. Within our PJI cohort, we observed a wide distribution of frailty, with mortality risk increasing nonlinearly as AFI-Core30 scores rise. This suggests that our frailty measure provides prognostic information beyond basic comorbidity counts. These findings agree with recent PJI studies recognizing frailty and nutrition as critical factors, with Wilson et al. showing that patients who are both frail and malnourished face substantially higher mortality risk. The combination of hypoalbuminemia and frailty yields a 23.3% positive predictive value and a likelihood ratio of 8.52 for inpatient mortality in hip fracture patients [14]. Such results highlight the importance of a comprehensive frailty assessment in PJI patients, as our AFI-Core30's ability to capture the full spectrum of health deficits allows for more accurate risk stratification than traditional, comorbidity-based methods. Ultimately, this supports more personalized treatment decisions.

The interpretation of mortality risk in PJI patients arises from multiple interconnected sources rather than a single cause. The systemic effects of infection, including sepsis, bacteremia, and potential organ dysfunction, directly contribute to mortality, especially in the acute phase [[15], [16], [17]]. Indirect complications further increase this risk through prolonged immobility leading to thromboembolism, nosocomial infections, and the cascade of medical decompensation that often follows major illness in vulnerable patients [18]. Underlying patient frailty is the third component, reflecting diminished physiologic reserve and a reduced capacity to withstand both the infection burden, surgical stress, and the additional indirect complications of PJI. This multifactorial mortality risk creates a complex clinical decision-making scenario where surgical aggressiveness must be carefully balanced. For patients with lower frailty scores, aggressive 2-stage exchange may theoretically offer advantages in infection eradication, as complete pathogen clearance remains a priority when the infection itself represents the primary mortality threat. Conversely, in highly frail patients, while persistent infection contributes to mortality, their limited physiologic reserve may heighten their risk from postoperative complications worsened by their increased preoperative frailty. In these cases, highly frail patients lacking immunosuppression and with identifiable pathogenic organisms responsible for the PJI might be considered for single-stage revision as a strategy to minimize operative trauma and avoid prolonged immobilization, though the optimal surgical approach in these populations warrants prospective investigation [19,20]. These patients may benefit from single-stage revision, which optimizes survival by minimizing operative trauma and avoiding prolonged immobilization [19,20]. Our model offers the mortality baseline against which clinicians can evaluate these competing risks, but treatment decisions must be individualized based on whether a patient is more likely to benefit from aggressive infection control or conservative management considering their overall health trajectory.

Beyond treatment selection, the risk stratification framework presented in this study can guide perioperative and postdischarge management plans. Patients identified as high 5-year risk may benefit from geriatric comanagement and proactive optimization of medical issues before elective reimplantation, along with closer follow-up that includes more frequent clinic visits, physical therapy to prevent functional decline, prehabilitation programs, and nutritional monitoring. Conversely, low-risk patients can safely adhere to standard pathways without additional interventions, provided that infection is properly managed. This framework may promote multidisciplinary management to stabilize comorbidities and support the selective referral of high-risk patients to specialized centers with expertise in complex PJI and geriatric care. Ultimately, this personalized risk stratification approach allows for individualized care pathways that tailor intervention intensity to each patient’s specific mortality risk, potentially enhancing both clinical outcomes and resource utilization in PJI management.

This study has several key strengths, including comprehensive model reliability assessments with calibration using 3 standard techniques (Platt scaling, isotonic regression, and beta calibration), all producing similar results with differences in Brier score of only 0.001-0.002, ensuring accurate mortality risk percentage predictions. We also addressed class imbalance and overfitting by applying inverse-prevalence weighting and cross-validation for complexity tuning, with consistent performance metrics between training and validation sets, indicating minimal overfitting. However, some significant limitations should be noted. As a registry-based study, clinical information was derived from diagnosis codes without case-specific granularity, including surgical technique, procedural staging, or microorganism profile. This limits the ability of the model to make definitive clinical recommendations regarding operative approach, and any such considerations should be interpreted as hypothesis-generating rather than evidence-based conclusions supported by these data. The AFI-Core30 was derived from available TriNetX data and may not capture every aspect of frailty, especially social support deficits that are difficult to code. Furthermore, our focus on all-cause mortality, while clinically relevant, does not encompass other important outcomes such as functional recovery, quality of life, or infection eradication success, which are also significant to patients and may involve different risk profiles than mortality alone. Additionally, the exclusion of native-joint septic arthritis codes in favor of prosthesis-specific diagnosis codes reflects a deliberate specificity-focused design choice; however, this may result in modest underestimation of the true PJI population, and future studies combining septic arthritis codes with total joint arthroplasty procedure codes should evaluate whether a sensitivity-based approach improves case ascertainment without sacrificing cohort homogeneity. Nonetheless, the findings of this study represent the first large-scale, frailty-based prognostic interactive model for long-term mortality after PJI, offering clinicians individualized risk estimates that can enhance patient counseling and treatment planning, shifting from subjective assessments to evidence-based decision-making in this complex clinical context.

## Conclusions

This study shows that age and frailty status, measured by the AFI-Core30, independently predict 5-year mortality in PJI patients. Mortality risk varies from 2.6% in young, healthy patients to 50.4% in older, frail individuals. The validated prognostic model has excellent calibration (Brier score 0.103), allowing clinicians to give evidence-based mortality risk estimates. These estimates can guide treatment choices, perioperative strategies, and shared decision-making. By using 2-dimensional contour maps and an interactive 3-dimensional surface plot to quantify individual mortality risk, this framework shifts PJI prognostication from subjective judgment to objective risk stratification. It could support more personalized care that aligns intervention intensity with patient-specific risk profiles. These findings are the first large-scale, frailty-based mortality prediction model for PJI patients and lay the groundwork for better clinical outcomes and resource use in PJI management.

## Declaration of generative AI and AI-assisted technologies in the writing process

During the preparation of this work, the author(s) used Claude Sonnet 4.5 (Anthropic, San Francisco) in order to improve readability and language. After using this tool/service, the author(s) reviewed and edited the content as needed and take(s) full responsibility for the content of the published article.

## CRediT authorship contribution statement

**Kole Joachim:** Writing – original draft, Methodology, Formal analysis, Data curation, Conceptualization. **Brandon Gettleman:** Writing – review & editing, Writing – original draft, Conceptualization. **Joshua Wiener:** Writing – review & editing, Data curation, Conceptualization. **Christopher Hamad:** Writing – review & editing, Conceptualization. **Adrian Lin:** Writing – review & editing, Conceptualization. **Amanda Perrotta:** Writing – review & editing. **Sumin Jeong:** Writing – review & editing. **Othneil Sparks:** Writing – review & editing. **Ezekiel Dingle:** Writing – review & editing. **Alexandra Stavrakis:** Writing – review & editing, Supervision. **Alexander B. Christ:** Writing – review & editing, Supervision.

## Conflicts of interest

A.B. Christ is a paid consultant for Stryker, Daiichi Sankyo, Smith & Nephew, Onkos Surgical, Globus Medical, and Zimmer Biomet Holdings; serves on the AAOS Hip & Knee Evaluation Committee, MSTS Fellowship Committee, and is the president of the ORS Implant Section; and also serves on the editorial or governing board of *Arthroplasty*. A. Stavrakis is a paid consultant for Smith & Nephew and Zimmer; all other authors declare no potential conflicts of interest.

For full disclosure statements refer to https://doi.org/10.1016/j.artd.2026.102090.

## Appendix A Supplementary data

Conflict of Interest Statement for Christ

Conflict of Interest Statement for Lin

Conflict of Interest Statement for Perrotta

Conflict of Interest Statement for Sparks

Conflict of Interest Statement for Stavrakis

Conflict of Interest Statement for Dingle

Conflict of Interest Statement for Gettleman

Conflict of Interest Statement for Hamad

Conflict of Interest Statement for Jeong

Conflict of Interest Statement for Joachim

Conflict of Interest Statement for Wiener
