# Supplementary material for: Predicting 5-Year Mortality After Prosthetic Joint Infection Using a 30-Item Accumulated Frailty Index in a 13,371-Patient Multicenter Cohort
Source: Arthroplast Today. 2026 Sep 4;41:102090. doi: 10.1016/j.artd.2026.102090 (PMC13571269; doi:10.1016/j.artd.2026.102090)
Supplement: Supplementary file 4 [file mmc12.docx]

| **Procedure** | **Type** | **Codes** |
| --- | --- | --- |
| THA | ICD-10-PCS | 0SR9019; 0SR901A; 0SR901Z; 0SR90J9; 0SR90JA; 0SR90JZ; 0SR90K9; 0SR90KA; 0SR90KZ; 0SRB019; 0SRB01A; 0SRB01Z; 0SRB0J9; 0SRB0JA; 0SRB0JZ |
| THA | CPT | 27130; 27132; 27134; 27137; 27138 |
| TKA | ICD-10-PCS | 0SRC0J9; 0SRC0JA; 0SRC0JZ; 0SRD0J9; 0SRD0JA; 0SRD0JZ |
| TKA | CPT | 27447 |

**Supplemental Table S1. Procedure Codes Used to Define Primary THA and TKA**
