# Supplementary material for: Predicting 5-Year Mortality After Prosthetic Joint Infection Using a 30-Item Accumulated Frailty Index in a 13,371-Patient Multicenter Cohort
Source: Arthroplast Today. 2026 Sep 4;41:102090. doi: 10.1016/j.artd.2026.102090 (PMC13571269; doi:10.1016/j.artd.2026.102090)
Supplement: Supplementary file 5 [file mmc13.docx]

| **Component** | **Description** | **Code System** | **Codes (as used)** |
| --- | --- | --- | --- |
| Immunosuppressed | Transplant, immunodeficiency, active immunosuppressive conditions | ICD-10  /ICD-9 | B20,B97.35,Z21,C77.0,C77.1,C77.2,C78.0,C78.1,C78.2,Z51.11,Z51.12,Z94.0,Z94.1,Z94.2,Z94.3,Z94.4,Z94.5,T86.10,T86.20,T86.30,D80.0,D80.1,D80.2,D80.3,D80.4,D80.5,D81.0,D81.1,D81.2,D82.0,D82.1,D82.2,D84.0,D84.1,D84.8,D84.9,E89.40,E89.41,M05.9,M06.9,M32.9,K50.9,K51.9,Z79.52,Z79.899 |
| Heart Failure (CHF) | Clinical heart failure codes | ICD-10/  ICD-9 | I50.1,I50.20,I50.21,I50.22,I50.23,I50.30,I50.31,I50.32,I50.33,I50.40,I50.41,I50.42,I50.43,I50.9,428.0,428.1,428.20,428.21,428.22,428.23 |
| COPD or Asthma | COPD and asthma codes | ICD-10/  ICD-9 | J44.0,J44.1,J44.9,J45.0,J45.1,J45.20,J45.21,J45.30,J45.31,J45.32,J45.40,J45.41,491.0,491.1,491.20,491.21,492.0,492.8,493.00 |
| Chronic Kidney Disease | CKD including ESRD/dialysis | ICD-10  /ICD-9 | N18.1,N18.2,N18.3,N18.4,N18.5,N18.6,N18.9,Z99.2,585.1,585.2,585.3,585.4,585.5,585.6,585.9 |
| Diabetes | Mixed type 1/2 diabetes definitions | ICD-10/  ICD-9 | E10.10,E10.11,E10.65,E10.69,E10.8,E10.9,E11.00,E11.01,E11.10,E11.11,E11.65,E11.69,E11.8,E11.9,250.00,250.01,250.02,250.03 |
| Coronary Disease / MI | Angina, MI, ischemic heart disease | ICD-10  /ICD-9 | I20.0,I20.1,I20.8,I20.9,I21.01,I21.02,I21.09,I25.10,I25.110,I25.111,I25.118,I25.119,410.00,410.01,410.10,410.11,414.00,414.01 |
| Atrial Fibrillation | Atrial fibrillation/flutter | ICD-10/  ICD-9 | I48.0,I48.1,I48.2,I48.91,427.31 |
| Stroke or TIA | Acute ischemic/hemorrhagic cerebrovascular disease, TIAs | ICD-10/  ICD-9 | G45.0,G45.1,G45.2,G45.8,G45.9,I63.00,I63.011,I63.012,I63.10,I63.20,434.00,434.01,434.10,434.11,435.0,435.1 |
| Peripheral Vascular Disease | Peripheral arterial disease and embolism | ICD-10/  ICD-9 | I70.20,I70.201,I70.202,I70.203,I70.208,I70.209,I70.21,I70.211,I70.212,I70.213,I70.218,I70.219,I70.22,I70.221,I70.222,I70.223,I70.228,I70.229,I70.23,I70.231,I70.232,I70.233,I70.238,I70.239,I70.24,I70.241,I70.242,I70.243,I70.248,I70.249,I70.25,I70.261,I70.262,I70.263,I70.268,I70.269,I70.29,I70.291,I70.292,I70.293,I70.298,I70.299,I70.8,I70.90,I70.91,I70.92,I73.00,I73.01,I73.1,I73.8,I73.9,I74.2,I74.3,I74.4,I74.5,I74.8,I74.9,E08.51,E08.52,E08.59,E09.51,E09.52,E09.59,E10.51,E10.52,E10.59,E11.51,E11.52,E11.59,E13.51,E13.52,E13.59,I70.92,I70.22,I70.24,440.20,440.21,440.22,440.23,440.24,440.29,440.30,440.31,440.32,440.8,440.9,443.8,443.9,444.21,444.22,444.81,250.70,250.71,250.72,250.73 |
| Cirrhosis or Advanced Liver Disease | Cirrhosis/hepatic failure spectrum | ICD-10/  ICD-9 | K70.30,K70.31,K71.0,K71.10,K71.11,K74.0,K74.1,K74.2,K74.3,K74.4,K74.5,K74.60,571.0,571.1,571.2,571.5,571.6 |
| Dementia | Degenerative and vascular dementias | ICD-10/  ICD-9 | F01.50,F01.51,F02.80,F02.81,F03.90,F03.91,G30.0,G30.1,G30.8,G30.9,G31.01,G31.09,290.0,290.10,290.11,290.20,290.21,331.0 |
| Depression or Anxiety | Depressive and anxiety disorders | ICD-10/  ICD-9 | F32.0,F32.1,F32.2,F32.3,F32.9,F33.0,F33.1,F33.2,F33.3,F41.0,F41.1,F41.8,F41.9,296.20,296.21,300.00,311 |
| Active Malignancy | Solid/hematologic malignancies | ICD-10 | C00–C97 |
| Low Albumin | Hypoalbuminemia | ICD-10/  ICD-9 | E88.09,R77.0,273.8,790.6 |
| Anemia | Anemia | ICD-10/  ICD-9 | D64.9,D64.0,D63.1,D50.9,D51.9,D52.9,D62,285.9,285.1,280.9,281.9 |
| Hyponatremia | Hyponatremia | ICD-10/  ICD-9 | E87.1,E87.0,276.1,276.0 |
| Lymphopenia | Lymphocyte deficiency | ICD-10/  ICD-9 | D72.810,D72.819,D72.820,D72.821,288.50,288.51 |
| Poor Glycemic Control | Diabetes with hyperglycemia / abnormal glucose | ICD-10/  ICD-9 | E10.65,E11.65,E13.65,R73.9,250.02,250.03,790.29 |
| Body Mass Index (BMI) | BMI extremes (underweight or severe obesity) from diagnosis | ICD-10/  ICD-9 | UNDERWEIGHT: R63.6,E43,E44.0,E44.1,783.22,263.9,263.0; BMI 40–59.9: E66.01,E66.2,E66.9,278.01; BMI ≥60: E66.3,278.03 |
| Chronic Opioids | From medication_afi_scores.csv (opioid chronicity score) | RxNorm | 7052,4337,7804,7676,7238,2670,8001,7814,3423 |
| Benzodiazepines / Antipsychotics | From medication_afi_scores.csv (sedative/antipsychotic score) | RxNorm | 2597,2356,596,2623,6470,11154,8076,89013,35636,41996,61381 |
| Hospitalizations (180 d) | Inpatient encounters in 180 days pre-PJI | Type IMP (inpatient) | — |
| ED Visits (180 d) | Emergency visits in 180 days pre-PJI | Type EMER (ED) | — |
| Falls | Documented falls or fall risk | ICD-10/  ICD-9 | W00,W01,W02,W03,W04,W05,W06,W07,W08,W09,W10,W11,W12,W13,W14,W15,W16,W17,W18,W19,Z91.81,E880,E881,E882,E883,E884 |
| Mobility Aids / DME | Wheelchair/cane/walker/ventilator dependence | ICD-10 | Z46.81,Z46.89,Z97.11,Z97.12,Z97.13,Z97.14,Z97.15,Z97.16,Z99.81,Z99.89 |
| Nursing Facility Residence | Long-term care or assistance | ICD-10 | Z59.3,Z74.01,Z74.09 |
| Tachycardia | Tachycardia or supraventricular tachycardia | ICD-10/  ICD-9 | R00.0,I47.1,I47.2,785.0 |
| Abnormal Blood Pressure | Hypertension crises, pulmonary HTN, hypotension, abnormal BP | ICD-10/  ICD-9 | I10,I11.0,I11.9,I12.0,I12.9,I13.0,I13.10,I13.11,I13.2,I15.0,I15.1,I15.2,I15.8,I15.9,I16.0,I16.1,I16.9,O10.01,O10.02,O10.03,O10.11,O10.12,O10.13,401.0,401.1,401.9,402.00,402.01,402.10,402.11,402.90,402.91,403.00,403.01,403.10,403.11,403.90,403.91,404.00,404.01,404.02,404.03,404.10,404.11,404.12,404.13,404.90,404.91,404.92,404.93,405.01,405.09,405.11,405.19,405.91,405.99,I27.0,I27.20,I27.21,I27.22,I27.23,I27.24,I27.29,I95.0,I95.1,I95.2,I95.3,I95.8,I95.9,458.0,458.1,458.2,458.8,458.9,R03.0,R03.1 |
| Hypoxemia (proxy) | Hypoxemia or acute/chronic respiratory failure | ICD-10/  ICD-9 | R09.02,J96.01,J96.02,J96.11,J96.12,799.02,518.81,518.82 |
| Unintentional Weight Loss/Cachexia | Weight loss, cachexia, failure to thrive | ICD-10/  ICD-9 | R63.4,R64,783.21,799.4 |

**Supplemental Table S2. AFI-Core30 Code Definitions**
