# Supplementary material for: Predicting 5-Year Mortality After Prosthetic Joint Infection Using a 30-Item Accumulated Frailty Index in a 13,371-Patient Multicenter Cohort
Source: Arthroplast Today. 2026 Sep 4;41:102090. doi: 10.1016/j.artd.2026.102090 (PMC13571269; doi:10.1016/j.artd.2026.102090)
Supplement: Supplementary file 6 [file mmc14.docx]

**Supplemental Figure S2.** [**Interactive 3D Surface Plot for 5-Year Mortality Prediction Stratified by Age and AFI-Core30 Scores**](https://tjaquadrant.github.io/AFI-Core30MortalityPrediction/afi_age_mortality_surface_updated.html)
