# Supplementary material for: Predicting 5-Year Mortality After Prosthetic Joint Infection Using a 30-Item Accumulated Frailty Index in a 13,371-Patient Multicenter Cohort
Source: Arthroplast Today. 2026 Sep 4;41:102090. doi: 10.1016/j.artd.2026.102090 (PMC13571269; doi:10.1016/j.artd.2026.102090)
Supplement: Supplementary file 13 [file mmc8.docx]

CONFLICT OF INTEREST STATEMENT

American Association of Hip and Knee Surgeons

(Adopted from the American Academy of Orthopaedic Surgeons disclosure statement)

The following form must be filled out completely and submitted by each author (example, 6 authors, 6 forms). If no discloser is required, please write/type ^“^none^”^ at the end of each sentence.

Manuscript Title: **Predicting 5-Year Mortality After Prosthetic Joint Infection Using a 30-Item Accumulated Frailty Index in a 13,371-Patient Multicenter Cohort**

1. Royalties from a company or supplier (The following conflicts were disclosed) **None**
2. Speakers bureau/paid presentations for a company or supplier (The following conflicts were disclosed) **None**

3A.	Paid employee for a company or supplier (The following conflicts were disclosed) **None**

3B.	Paid consultant for a company or supplier (The following conflicts were disclosed) **None**

3C.	Unpaid consultants for a company or supplier (The following conflicts were disclosed) **None**

4. Stock or stock options in a company or supplier (The following conflicts were disclosed) **None**
5. Research support from a company or supplier as a Principal Investigator

(The following conflicts were disclosed) **None**

6. Other financial or material support from a company or supplier (The following conflicts were disclosed) **None**
7. Royalties, financial or material support from publishers (The following conflicts were disclosed) **None**
8. Medical/Orthopaedic publications editorial/governing board (The following conflicts were disclosed) **None**
9. Board member/committee appointments for a society (The following conflicts were disclosed) **None**

EachauthormustsignANDprintortypehis/hername,dateandsubmitaseparateform.

In addition, one BLINDED Conflict of Interest form (no author names used) should be submitted per manuscript with all author disclosures.

Christopher Hamad	Christopher Hamad	November 12, 2025

Author Name (Print or Type)	Author Signature	Date
